# Molecular Arrangement in Self-Assembled Azobenzene-Containing Thiol Monolayers at the Individual Domain Level Studied through Polarized Near-Field Raman Spectroscopy

**DOI:** 10.3390/ijms12021245

**Published:** 2011-02-21

**Authors:** Marc Chaigneau, Gennaro Picardi, Razvigor Ossikovski

**Affiliations:** 1 LPICM, Ecole Polytechnique, CNRS, 91128 Palaiseau, France; E-Mails: marc.chaigneau@polytechnique.edu (M.C.); gennaro.picardi@dcu.ie (G.P.); 2 NCSR, School of Chemical Sciences, Dublin City University, Dublin 9, Ireland

**Keywords:** raman spectroscopy, TERS, azobenzene, polarization

## Abstract

6-[4-(phenylazo)phenoxy]hexane-1-thiol self-assembled monolayers deposited on a gold surface form domain-like structures possessing a high degree of order with virtually all the molecules being identically oriented with respect to the surface plane. We show that, by using polarized near-field Raman spectroscopy, it is possible to derive the Raman scattering tensor of the ordered layer and consequently, the in-plane molecular orientation at the individual domain level. More generally, this study extends the application domain of the near-field Raman scattering selection rules from crystals to ordered organic structures.

## 1. Introduction

Raman spectroscopy is an extremely useful and widespread technique for material characterization [1,2]. Apart from offering distinctive spectral signatures allowing for the rapid identification of the chemical nature of the sample, even more detailed information on the molecular (or crystalline) order can be retrieved by studying the signal dependence on the polarization of the two involved photons (incident and scattered), as formally defined by the Raman scattering tensor [3]. Thus, domain orientation in crystals can be effectively assessed by polarized Raman spectroscopy featuring high sensitivity and spatial resolution (few hundreds of nanometers) while requiring small sample volumes and little, if any, sample preparation [4,5]. Thus, Raman polarization studies have been performed to determine the orientation [6,7] and structure [8] of molecular thin films, a required characterization step for the deposition of organic films to be used in technical applications such as organic light emitting diodes, flat panel displays and thin film transistors [9].

Further, higher spatial resolution, below the diffraction limit, can be attained by taking advantage of near-field optical techniques. The best performing approach for Raman spectroscopy is the aperture-less configuration [10] in which the local electromagnetic field generated upon light scattering on a metal tip apex can be strongly enhanced by a localized surface plasmon excitation. Within such Tip Enhanced Raman Spectroscopy (TERS) [11], an Au or Ag tip kept at nanometer distance from the sample surface is externally illuminated and the Raman signal from the sample region directly below the tip, usually dominating the undesired far-field background, is collected. The lateral resolution of TERS is thus not related to the exciting laser spot size but rather to the physical dimensions of the tip apex (typically less than 50 nm) [12–14]. Noticeably, the metal tip not only enhances the local field, and consequently, the Raman scattering process in the analyte, but also modifies the polarization state of both the exciting and scattered photons. This is due to a preferential enhancement of the field component parallel to the tip axis (*i.e.*, the surface-normal component) [15,16]. This “polarization sensitivity” property has been proposed as a possible way to discriminate between near-field and far-field components when probing crystalline materials, which present a strongly polarized Raman response [17–19]. Our group proposed a phenomenological model to account for the polarization dependence of the TERS signal from crystalline silicon based on the introduction of the concept of tip amplification tensor [20]. Recently, this formalism has been adopted to spatially resolve ferroelectric domains in individual BaTiO_3_ nanocrystals, introducing the concept of a nanocrystollography based on near-field Raman selection rules [21]. Clearly, it would be interesting to extend the application fields of TERS to thin organic layers whenever these exhibit, at least locally, an ordered, crystalline-like structure.

Chemical modification of a planar gold surface with molecules exhibiting a thiol anchor group leads to the formation of highly ordered Self Assembled Monolayers (SAM), in which virtually all the anchored molecules are equally tilted with respect to the surface normal [22,23]. For the 6-[4-(phenylazo)phenoxy]hexane-1-thiol (an azobenzene-containing thiol) SAMs, an in-plane order is further established due to the interaction between the phenyl rings of neighboring units. While reflection-absorption infrared spectroscopic and ellipsometric measurements confirmed a nearly perpendicular orientation of the azobenzene moieties [24–26], scanning probe microscopy studies revealed the presence of small domains having the azobenzene groups arranged in regular lattice structures. The formation of such domains is possible due to the flexibility of the (sulfur terminated) alkyl chain linking the aromatic system to the surface (so-called bundle model) [27–31]. This makes azobenzene derivative SAMs particularly interesting for polarized TERS studies to test the capability of Raman spectroscopy in the near-field optical regime while aiming at the determination of the crystalline domain orientation with sub-diffraction limited lateral resolution. Surface enhanced Raman spectroscopy (SERS) has also been used to report on the monolayer formation of the azobenzene-containing thiol on electrochemically roughened gold electrodes or on nanostructured substrates formed by electron beam lithography [32,33].

## 2. Experimental Section

Our TERS set-up consists of a confocal Raman microscope (Labram HR800, HORIBA Jobin Yvon) with a 633 nm HeNe laser source optically coupled in an oblique backscattering geometry to a scanning probe microscope or SPM (XE-100, Park Systems). The long-working-distance objective (50×, NA = 0.45) is oriented at 65° with respect to the sample normal and produces an elliptical laser spot with approximate axis lengths 2.5 μm × 1 μm. Note that in the oblique incidence (off-axis) backscattering geometry, the scattering plane is defined by the direction of the incident/scattered radiation and the sample normal (coinciding with the tip axis). The opto-mechanical coupling is motorized along the *x*, *y* and *z* axes and allows for the accurate alignment of the exciting light spot with respect to the tip apex. The SPM is operated in scanning tunneling microscopy (STM) mode; the tips used were prepared from Au wire pieces of 250 μm diameter and 99.99% purity (from Goodfellow) by electrochemical etching in a concentrated HCl/ethanol 1:1 solution according to an existing procedure [34]. Tunneling parameters were 100 mV bias voltage, tip positive, and 40–30 pA currently.

The polarization state of the incident beam can be continuously varied between 0° (*s* polarization or *s*-pol; electric field perpendicular to the scattering plane) and 90° (*p* polarisation or *p*-pol; electric field parallel to the scattering plane) by using a half-wave plate in a motorized rotating mount (0.5° azimuth uncertainty) inserted in the excitation laser beam path. During the TERS experiments, laser power was kept below 0.5 mW at the sample surface to avoid any possible heating and “bleaching” side-effects.

The atomically flat Au(111) substrates were purchased from Arrandee. Synthesis of the 6-[4-(phenylazo)phenoxy]hexane-1-thiol closely followed the procedure reported in [30]. A 5 mM solution in ethanol was used for the monolayer absorption on the freshly flame annealed Au(111) substrates. An absorption time of 6 h proved to be sufficient to reach full coverage [35]. After removal from the solution, the sample was rinsed in pure ethanol and distilled water, and dried with N_2_.

## 3. Results and Discussion

**Figure 1.**
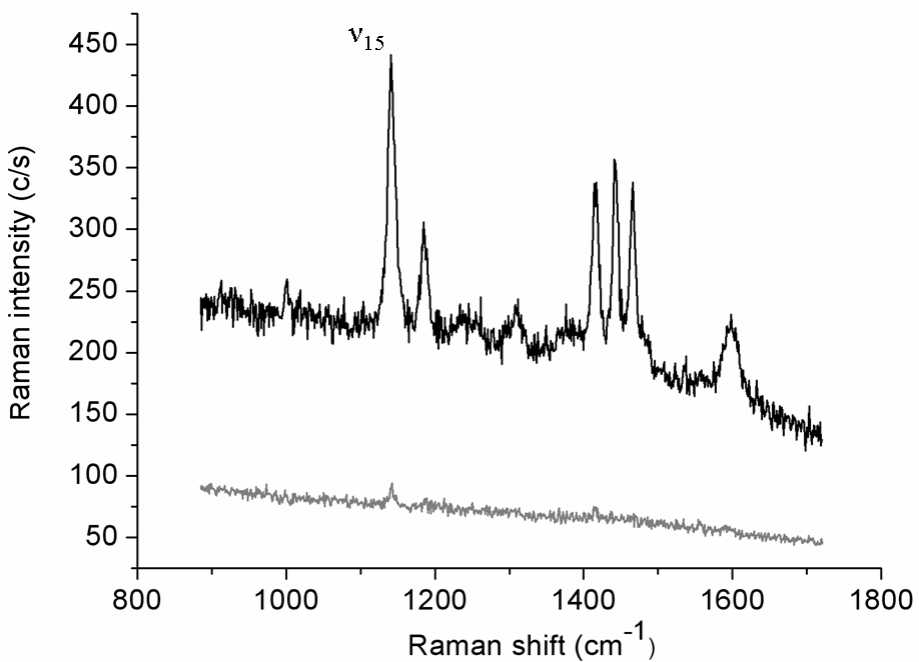
Normal (far-field) and tip-enhanced Raman spectra of the azobenzene thiol monolayer adsorbed on a Au(111) substrate. Lower curve: normal spectrum (tip-retracted); upper curve: tip-enhanced spectrum (tip-in-tunneling mode).

Polarized TERS experiments were mainly conducted on smooth regions of the supporting metal film: Adsorption at an atomically flat surface is needed for the formation of the ordered thiol layer. Before starting the spectral acquisitions, the topography of the surface was systematically checked through STM mode mapping and the tip was moved over a flat region. The observed Au(111) terraces typically size up to few hundreds of nm [35]. The lower spectrum in Figure 1 reports the Raman spectrum (lower curve) collected in 30 s (0.5 mW incident laser power; *p-*pol) from an azobenzene-containing thiol layer adsorbed onto the gold film. This weak-intensity signal originates from the whole illuminated spot area while the tip is retracted few microns above the surface. The upper spectrum (1 s acquisition time) is relative to the same illuminated sample area but with the gold tip in tunneling mode. The estimated tunneling gap distance between the underlying gold surface and the tip is ∼3 nm, including the 2 nm-thick organic layer [35]. The tip-down-tip-up contrast ratio derived from the two spectra is about 10. The far-field elliptical spot area is approximately 7 μm^2^ and the tip radius produced by the etching procedure is usually ∼30 nm, as estimated from SEM images. If considering to a first instance the circular area directly below the tip apex as uniformly affected by the enhanced field and a uniform coverage of the thiol layer, a TERS enhancement factor of the order of 1 × 10^4^ can be estimated. Similar absolute signal levels (not shown) and contrast ratios could be obtained on several different regions of the sample surface. Six major spectral features assigned to the vibrations of the anchored double-ring system in a *trans* configuration [36,37] are clearly seen in the TERS spectrum. More specifically, all bands observed correspond to in-plane vibrations with A_g_ symmetry that mostly couple with the surface-normal local electric field component.

Next, the intensity of the strongest peak (ν_15_) at 1141 cm^−1^, assigned to the in-phase stretching of the two C–N bonds, was monitored while changing stepwise the incident polarization from *s*- to *p*-pol and back to *s*-pol, for both the far-field (tip retracted) and the near-field (tip tunneling) cases. Before discussing the resulting polarization curves, we shall describe the relations necessary for modeling the polarized near-field Raman scattering experiment.

The influence of the incident light polarization on the intensity of the Raman signal scattered by the thiol layer in the presence of the local field enhancing tip can be quantitatively described by adopting the same model we originally proposed and tested on bulk crystalline samples [20]. In the following, only the basic relationships will be recalled; more details on the simulation procedure can be found in [20,38]. Within the classical formalism of normal (*i.e.*, non-resonant) Raman scattering [1,2], the far field scattered intensity is given by the well-known selection rule

(1) $$I_{\text{ff}}=K{\left|{\text{e}}_{s}^{\;T}\;\text{R}\;\;{\text{e}}_{e}\right|}^{2}$$

in which *K* is a proportionality constant, **e***_e_* and **e***_s_* are the exciting (or incident) and scattered electric field vectors, respectively, and **R** is the Raman scattering tensor of the involved molecular (or crystal) vibration. Note that the matrix expression for the scattering tensor **R** entering Equation 1 depends on the reference frame used. In particular, if the sample is rotated about its normal, the tensor **R** will change accordingly; this change can be formally described by rotating the matrix of **R** by a rotation matrix. As a consequence, the scattered intensity given by Equation 1 depends not only on the polarization states **e***_e_* and **e***_s_* of the exciting and scattered radiations, but also on the sample azimuth (or orientation) through the tensor **R**.

To describe the near-field scattering process, a phenomenological tip-amplification tensor **A**, accounting for the enhancement of the local electric field by the tip, is introduced [20]. The tensor **A** expresses the fact that the electric field component parallel to the tip axis is preferentially amplified compared to that perpendicular to it. Its matrix representation in the tip-sample reference frame (with *x* and *y* axes perpendicular to the tip axis, *i.e.*, parallel to the sample plane; *z* axis along the tip axis) is

(2) $$\text{A}=\left(\begin{array}{ccc} b & 0 & 0\\ 0 & b & 0\\ 0 & 0 & a \end{array}\right)$$

in which *a* and *b* (with *a* > *b*) are phenomenological coefficients expressing the different amplification ratios of the electric field components parallel and perpendicular to the tip axis. The tip geometry (*i.e.*, form, radius of curvature, apex angle) and material dielectric constant determine the actual values of the amplification factors.

The near-field (tip-enhanced) scattered intensity *I_nf_* is given by

(3) $$I_{\text{nf}}=K^{\prime }\sum_{k}{\left|{\text{e}}_{s}^{\;T}\;{\text{R}}^{\prime }\;{\text{e}}_{e}\right|}^{2}$$

in which the near-field proportionality constant *K′* is generally different from *K*, the far-field one, and **R**′ is the modified (or effective) Raman scattering tensor taking into account the action of the tip. Within the near-field scattering process, the polarization state of the exciting radiation is first altered by the tip, then molecular vibrations excited by this enhanced field generate the scattered radiation, and finally a second tip-scattered-radiation interaction takes place. As a result, the expression for the effective scattering tensor **R**′ becomes

(4) $${\text{R}}^{\prime }={\text{A}}^{T}\;\text{R}\;\;\text{A}$$

in which **A** is the tip-amplification tensor from Equation 2.

Finally, the total scattered field intensity, *I_tf_*, measured in “tip down” position, is given by the sum of the far-field, Equation 1, and the near-field, Equation 3, contributions,

(5) $$I_{\text{tf}}=I_{\text{ff}}+I_{\text{nf}}$$

since the two signals superimpose incoherently on the detector.

The azobenzene molecule in its *trans* conformation belongs to the C_2h_ spatial point group [36], which results in a Raman scattering tensor with four independent components for A_g_-symmetry active modes. Taking the *z* axis along the surface normal, also corresponding to the molecular long axis, and identifying the *xz* plane as the molecular plane (*i.e.*, assuming an upright orientation of the azobenzene moieties), the Raman tensor **R**_Ag_ of a single azobenzene molecule has the general form

(6) $${\text{R}}_{\text{Ag}}=\left(\begin{array}{ccc} \alpha_{\text{xx}} & 0 & \alpha_{\text{xz}}\\ 0 & \alpha_{\text{yy}} & 0\\ \alpha_{\text{xz}} & 0 & \alpha_{\text{zz}} \end{array}\right)$$

As reported by Pedersen *et al.* [39] for the azobenzene chromophore in the *trans* configuration, both the absorption cross-section along the *y* axis and the polarizability tensor component normal to the molecular plane, *i.e.*, the *yy* component, are negligible at the exciting wavelength used in our experiments (note the different labeling of the molecular axes used here with respect to that of the cited reference). Keeping in mind that the Raman tensor is the spatial derivative of the polarizability tensor with respect to the normal mode vibrations (here, the ν_15_ mode), we assume the *yy* polarizability component to change only weakly with the ν_15_ molecular vibration analyzed here, allowing to simplify the above tensor form by setting α*_yy_* = 0 in Equation 6. Further, the Raman tensor for the monolayer may be obtained from a superposition of the Raman tensors of the single molecules within the unit cell of the type of lattice formed [40]. Azobenzene-containing thiols having different molecular structures may form different types of regular domains extending over several tens of nanometers: (a) domains of centered hexagonal lattice with a rectangular unit cell and two molecules per unit cell, producing herringbone structures, *i.e.*, alternate horizontal rows of canted molecules are formed by HS(CH_2_)_11_OC_6_H_4_N=NC_6_H_5_, having a longer anchoring tail than the azobenzene derivative studied here [27] and (b) domains with an (oblique) nearly rectangular lattice likewise having two molecules per unit cell are formed by HS(CH_2_)_6_OC_6_H_4_N=NC_6_H_5_ [28–31]. In this second case, the two molecules in the unit cell may be almost parallel or form an angle. For a parallel dimer the molecular lattice tensor retains the single-molecule form (6). In the second case of two molecules forming an angle of approximately 40° between their short molecular axes (the longer axis being along the *z* axis) [30], the resulting unit cell tensor is obtained by summing the individual tensors of the two molecules, the second tensor being rotated at the angle of 40° with respect to the first one,

(7) $${\text{R}}_{\text{Azo}}={\text{R}}_{\text{Ag}}+{\text{T}}^{T}(40{}^{\circ}){\text{R}}_{\text{Ag}}\;\text{T}(40{}^{\circ})$$

still leaving only two “free” parameters, α*_xx_* and α*_xz_* (the element α*_zz_* is set equal to unity because of the relative nature of the polarized TERS experiment and model). In Equation 7, the transformation matrix **T** (θ) is simply a rotation matrix about the *z* axis at the angle θ = 40°,

(8) $$\text{T}(\theta )=\left(\begin{array}{ccc} \text{cos}\theta & \text{sin}\;\theta & 0\\ -\text{sin}\;\theta & \text{cos}\theta & 0\\ 0 & 0 & 1 \end{array}\right)$$

The tensor form (7) is used in the modeling procedure described above to fit the “tip tunneling” response of the azobenzene derivative layer. To account for the various possible orientations of the domain lattice with respect to the scattering plane, **R***_Azo_* is further rotated about the *z* axis at the appropriate domain azimuth angle ϕ by using a transformation matrix of the same form as Equation 8; *i.e.*,

(9) $${\text{R}}_{\text{Azo}}(\phi )={\text{T}}^{T}(\phi ){\text{R}}_{\text{Azo}}\;\text{T}(\phi )$$

Considering that the TERS intensity comes from the highly localized region below the tip apex, it can be expected that its polarization-dependent response reflects the local domain structure and, furthermore, that it differs substantially from its far-field counterpart whereby a much larger area encompassing a large number of differently oriented domains with, possibly, disordered zones is probed.

**Figure 2.**
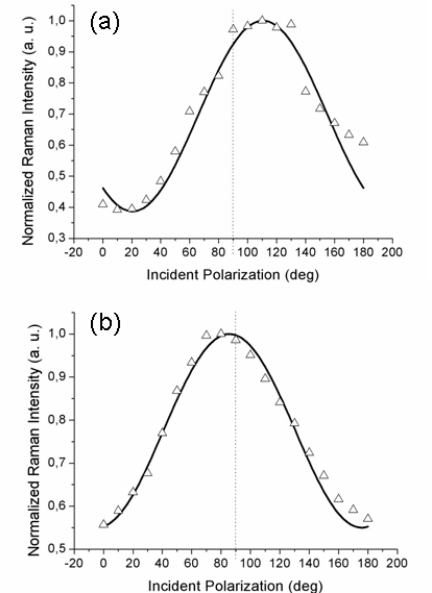
Normalized TERS intensity from the azobenzene containing thiol layer as a function of the incident light polarization collected at two tip positions, (**a**) and (**b**), on the sample. The shifts of the maximum signal intensity from incident *p*-polarization (90°) result from the different orientations of ordered molecular domains with respect to the scattering plane.

Figure 2a,b reports the experimental data obtained with the gold tip consecutively located at two positions on the surface separated by approximately 300–400 nm; note that there was no analyzer in the output beam (so-called “depolarized” or, more correctly, unanalyzed scattering configuration). With 1 s integration time for each TERS acquisition, a complete polarization curve takes about 20 s to be obtained. Considering the thermal drift of our set-up (0.1 nm/s), the tip creeps negligibly over the organic layer during this time.

It is to be noted that the maximum of the TERS signal is observed at two different values of the incident polarization, both slightly shifted from the expected *p*-pol (represented by a dotted line in both figures): an upshift, for the curve in Figure 2a, and a downshift, for the curve in Figure 2b. Three other curves (not shown) recorded with different tips likewise showed a small (but non-negligible) shift of the maximum intensity position from the 90° value for incident *p*-polarization. This behavior is reproduced by our model by considering different azimuthal orientations ϕ of the probed molecular domain (described by the rotated **R***_Azo_*(ϕ) tensor; see Equation 9) with respect to the scattering plane. Indeed, the magnitudes and the signs of the shifts are functions of the domain azimuth angle reflecting the local orientation of the domains. The solid lines are the best fits obtained with the model described above for the set of component values α*_xx_* ≈ 0.5 and α*_xz_* ≈ 0.3 and at two different domain azimuth angles ϕ. The polarized tip-enhanced Raman signal is thus clearly shown to be sensitive to the local in-plane order. Note that the single molecule tensor form (6) also reproduces the experimental curves for different domain azimuth angles. Therefore, it is not possible, from these measurements, to distinguish between the two possible molecular orientations within the unit cell. It should be also noted that the possibility of the field-enhanced region to extend over few contiguous domains rather than a single one cannot be ruled out *a priori* and the retrieved values for the tensor components would then represent a local average.

**Figure 3.**
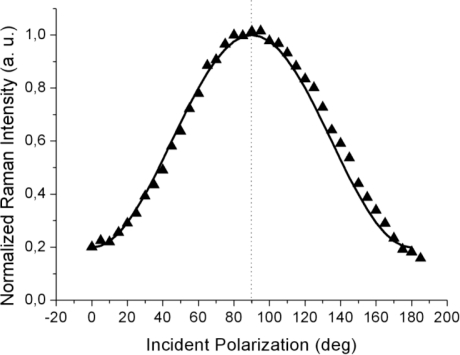
Normalized peak intensity in the far field (tip retracted) of the azobenzene derivative ν_15_ Raman mode as a function of the incident light polarization. The solid line shows the best fit to the data. The dotted line corresponds to *p*-polarization.

The far-field spectra shown in Figure 3 were acquired at higher laser power (5 mW) and at a much longer acquisition time (20 s per point) to attain a sufficient signal level, including for the weaker response to the incident *s-*polarization. In contrast to the TERS curves from Figures 2a,b, the far-field (tip retracted) response always peaked at 90° corresponding to *p*-polarization (shown by the dotted line; within the 3° azimuth offset of the half-wave plate fast axis) as can be seen from Figure 3. This fact clearly indicates that over the large illuminated area, most of the planar molecules chemisorbed at the metal surface have their long axes oriented parallel to the sample normal, but with no other detail regarding the presence of an in-plane order, *i.e.*, the presence of regular domains

To reproduce the far-field behavior (the solid line in Figure 3), the Raman tensor (9) was again used and the intensity contributions for four equally spaced domain azimuth angles (0°, 90°, 180° and 270°) were summed up incoherently to account for the random orientation of the domains and the possible presence of disordered zones within the large illuminated area. Indeed, over a μm-scale laser spot, a great number of ordered (nano-) domains are probed and their relative orientations remain spatially unresolved in the far-field response. As a result, the far-field polarization curve peaks at 90°, in accordance with the random domain orientation in the *xy* plane.

Comparison of the Raman signal intensity recorded at incident *s*-polarization (0° and 180° values in Figures 2 and 3) for the near-field and far-field cases shows that a non-negligible signal enhancement is also present for this polarization. Within the TERS community, it is usually accepted that a more efficient plasmonic excitation and, consequently, a greater tip enhancement are achieved with incident *p*-polarization (having a significant electric field component oriented along the tip axis) while almost no enhancement is expected for incident *s*-polarization [41]. Nonetheless, in a series of experiments performed on bulk crystalline materials, [42–44] it has been clearly shown that metal tips convert, to a certain extent, the incident field polarization from *s*- to *p*-pol. and *vice versa*. This “depolarization” (more correctly, cross-polarization) phenomenon is intrinsically related to the metal tip scattering, and may have also affected our measurements, although we believe only to a minor degree owing to the very small thickness of the thiol layer as compared to the micrometer penetration depth of visible light into bulk crystalline materials for which the phenomenon has been reported.

Also, a feature that is sometimes overlooked when considering the preferential tip enhancement for radiation having the electric field parallel to the tip axis is that the Raman process involves two photons: the polarization state of the incident photon is directly defined by the polarizer setting, while the polarization state of the Raman scattered photon further depends on the Raman scattering tensor. Depending on the tensor form (presence of non-zero off-diagonal elements), certain vibration modes can be (also) excited with *s*-polarized light and generate *p*-polarized light that will then be enhanced by the tip, the net effect resulting in a near-field signal enhancement with *s*-polarized excitation. This is exactly our case with **R***_Ag_* from (6)—and **R***_Azo_* from (7)—having α*_xz_* ≠ 0. This fact is most possibly responsible for the unexpectedly high TERS intensity recorded at incident *s*-polarization.

To further support the conclusion of the TERS polarization curves being mainly affected by the molecular order at a sub-micrometer length scale, we present the results obtained when probing (with the same tip) two regions on the sample surface having different local morphology of the underlying gold substrate.

**Figure 4.**
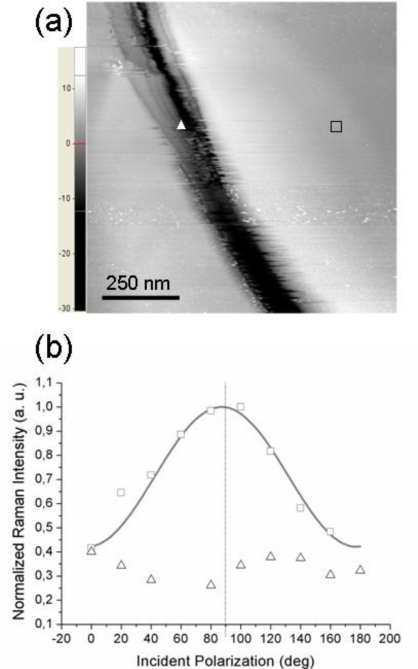
(**a**) STM image from a region of the azobenzene thiol covered Au(111) surface. TERS spectra were recorded with the tip located at two positions marked with a triangle and a square; (**b**) Normalized peak intensity of the azobenzene ν_15_ Raman mode as a function of the incident light polarization for the two positions shown in (**a**).

Figure 4a reports the STM topography of a sample region on which two distinct TERS measurements were performed: the tip was first located on the flat area near the trench (position marked with a square) and second, the tip was moved inside the depression (position marked with a triangle). The experimental points (squares and triangles) shown in Figure 4b refer to the corresponding TERS signal intensity. As readily seen, when the tip is located over a flat region, a well pronounced modulation of the detected signal by the incident polarization is observed (squares). Note that the curves in Figure 2a,b exhibit similar modulation amplitudes; however, in this case the signal intensity peaks quite close to 90° incident polarization (this particular value is obtained in the simulated near-field curve when the scattering plane accidentally coincides with the *xz* molecular plane). In contrast, the signal intensity recorded with the tip located inside the depression (triangles in Figure 4b) appears to be much less influenced by the incident polarization; moreover, its behavior is not a clearly sinusoid-like one, unlike the previous ones.

This situation may arise whenever the probed molecules do not have a well-defined orientation with respect to the tip axis, *i.e.*, when it is no more possible to define a unique substrate plane and the azobenzene molecular planes are more or less randomly oriented in space (but their responses still add coherently because of the local nature of the TERS signal). This is the situation whenever probing a very rough region of the gold substrate that cannot support the formation of a highly ordered molecular overlayer (having all molecular planes parallel to the tip axis). A TERS signal generated from the coherent contributions of the thiol molecules adsorbed at gold sites is still recorded but the short-range order is partially or even completely lost, with the disorder being inherently related to the topography of the supporting metal. Further, we need also to consider the local geometry of the gold tip—gold surface gap. With the tip located over a flat region, a well defined gap is formed between the lower (curved) surface of the tip apex and the opposite metal surface (the gap axis essentially coincides with the tip axis) whereas with the tip located inside a shallow trench, differently oriented gaps may be formed between the sides of the approximately spherical apex and the sloping surface. This picture points out the critical role played by the underlying gold surface in defining not only the magnitude of the field enhancement but also its influence on the orientation of the enhanced field. Remarkably, we have noted that by allowing the single-molecule tensor from Equation 6 to be tilted away from the *z* (tip) axis, one can also reproduce (for certain values of the tilt and azimuth angles) an almost flat polarization dependence curve (not shown), qualitatively similar to the TERS response from a rough sample region.

## 4. Conclusions

Azobenzene-containing thiol molecules self assembling on a Au(111) substrate can arrange in two-dimensional ordered (nano-)domains. The related in-plane arrangement and domain orientation affect the intensity of the Raman signal recorded at various incident polarizations, provided that the probed area has a size comparable to that of the domains. This was observed in polarized TERS measurements in which only the molecules located in the close proximity of the nanometer-sized tip apex contributed to the enhanced signal. We applied the tip amplification tensor model to describe the polarization response of the locally ordered monolayer, thus extending the validity of the model from crystal structures to ordered organic layers. A different behavior was observed when probing disordered regions on the sample surface and especially when micrometer-large areas were probed in the far field, whereby signal response averaging from several domains occurred, thus confirming the spatially localized nature of the TERS signal.
